# Transitions and Long-Term Clinical Outcomes in Patients Admitted in Intensive Care Units Receiving Continuous Renal Replacement Therapy

**DOI:** 10.3390/jcm13175085

**Published:** 2024-08-27

**Authors:** María Valdenebro, Jose Portoles, María Luisa Serrano Salazar, Ana Muñoz Sánchez, Ines Alameda-Aguado, Leyre Martín Rodriguez, Felipe Zalamea-Jarrin, Paula López-Sánchez

**Affiliations:** 1 Nephrology Department, Hospital Universitario Puerta de Hierro Majadahonda, IDIPHISA, 28222 Majadahonda, Spain; mdev183@hotmail.com (M.V.); mserranosalazar@gmail.com (M.L.S.S.); ams_1993@hotmail.com (A.M.S.); leyremartinrodriguez@gmail.com (L.M.R.); fezalamea@gmail.com (F.Z.-J.); nefro_metodologia@yahoo.com (P.L.-S.); 2 Medicine Department, Universidad Autónoma de Madrid, 28049 Madrid, Spain

**Keywords:** acute kidney injury, extracorporeal blood purification therapy, continuous venovenous hemodiafiltration, hemodynamic instability, mortality, critical care

## Abstract

**Introduction**: Acute kidney injury (AKI) significantly disrupts vital renal functions and is a common and serious condition in intensive care units (ICUs). AKI leads to extended hospital stays, increases mortality rates, and often necessitates nephrology consultations. Continuous renal replacement therapy (CRRT) plays a central role in managing AKI, requiring a multidisciplinary approach involving nephrologists, intensivists, and anesthesiologists. This study examines the clinical profile and progression of AKI in ICU patients requiring CRRT, with a focus on CRRT indications and modalities. **Materials and Methods**: We conducted a single-center retrospective observational study on ICU patients with AKI requiring CRRT from January to December 2019. AKI diagnosis followed the RIFLE criteria, and patients who received CRRT for less than 36 h were excluded. Data collected included demographics, hemodynamic parameters, and renal function parameters, with follow-ups at 1 week, 1 month, 6 months, and 12 months. Statistical analyses evaluated outcomes and transitions between CRRT and other renal replacement therapies. **Results**: Among 123 evaluated patients, 95 met inclusion criteria. Fifteen patients received CRRT for less than 36 h, with an early mortality rate of 80%. The final cohort comprised 80 patients who underwent CRRT for over 36 h, with a mean age of 65.3 years (SD = 13.6) and a Charlson index of 6.4. Patients were categorized based on primary diagnosis into heart failure, cardiac surgery, sepsis, other surgeries, and miscellanea groups. Mortality rates were highest in the heart failure and miscellanea groups. Significant variability was observed in therapy transitions and long-term outcomes. Continuous venovenous hemodiafiltration (CVVHDF) was the most frequently used CRRT modality. **Conclusions**: This study highlights the variability in CRRT practices and the poor prognosis for critically ill patients with AKI requiring CRRT. Timely nephrology consultation and tailored treatment plans may improve patient outcomes and optimize CRRT utilization. Future research should focus on refining CRRT protocols and exploring preventive strategies for AKI.

## 1. Introduction

Acute kidney injury (AKI) impairs the kidneys’ ability to eliminate toxins, regulate acid–base balance, and maintain electrolyte levels [1,2]. This condition, common in intensive care units (ICUs), prolongs hospital stays, increases mortality, and often necessitates nephrology consultations [3,4,5]. AKI in ICUs is frequently associated with multiple organ failure, requiring the in-depth knowledge of fluid and electrolyte homeostasis, as well as continuous renal replacement therapies (CRRTs) [6]. The Spanish FRAMI study reported an AKI incidence of 5.7%, which rises to 8.5% when coronary patients are excluded [7].

Since the 1990s, CRRT has become essential in managing AKI, especially given the unpredictable and varied etiologies of this condition in critically ill patients. Initially managed by anesthesiologists and intensivists, AKI management has evolved into a multidisciplinary approach that now includes nephrologists, who focus on preventive surveillance and nephroprotection. Effective AKI management in the ICU requires continuous care, encompassing early detection, timely intervention, and post-discharge follow-up. Despite technological advances, AKI continues to significantly worsen patient outcomes, with persistently high mortality rates [7,8,9,10].

Published clinical trials tend to focus on very specific aspects such as particular patient profiles, the timing of therapy indication, the techniques employed in de-escalation strategies, and risk factors associated with a poor prognosis. Clinical guidelines [11,12] and reviews are based on these studies, but they are not conclusive and may not fully capture the variability and complexity of real-world clinical settings [13].

In this scenario of complex patient management by multiple specialists and limited available evidence, we designed our study to analyze the practical realities of CRRT treatment in the ICU of a tertiary referral hospital, aiming to identify areas for improvement. This study describes the clinical profile and evolution of AKI in ICU patients requiring CRRT, with a detailed focus on the indications and modalities of CRRT. The findings highlight the critical importance of a collaborative, multidisciplinary approach to improving outcomes for critically ill patients with AKI.

## 2. Materials and Methods

A single-center retrospective observational study was conducted, which included all patients admitted in the ICU with AKI requiring CRRT between January 2019 and December 2019. AKI diagnosis was made according to the Risk Injury Failure Loss End Stage Renal Failure (RIFLE) criteria by the Acute Dialysis Quality Initiative (ADQI) [11]. For patients with unknown baseline serum creatinine (sCr) levels and no chronic kidney disease (CKD) history, an sCr value > 1.5 mg/dL with diuresis < 0.5 mL/kg/h indicated AKI. We also applied the Kidney Disease Improving Global Outcomes (KDIGO) [12] criteria to stage AKI from levels 1 to 3. At the initiation of CRRT, all cases were classified as “Failure” according to the RIFLE criteria and AKI stage 3.

Our objective was to describe the profiles of patients undergoing CRRT and to assess both short- and long-term outcomes, with particular focus on the transitions between different CRRT techniques in a real-world clinical setting. From this analysis, we aim to propose areas for healthcare improvement that are tailored to the realities of our comprehensive public health system.

To achieve our primary objectives, we excluded patients who received CRRT for less than 36 h and established a cut-off point at the end of the first CRRT period. This allowed for a more precise analysis of technique transitions and clinical outcomes. We evaluated these transitions, along with renal and patient survival, at 1 and 4 weeks, as well as at 6 and 12 months following this initial cut-off point.

We classified patient profiles based on primary diagnosis rather than on the reason for CRRT indication. This led to the definition of the following five groups for the surgical and medical ICUs: heart failure: patients with acute pulmonary edema or refractory fluid overload following acute myocardial infarction, arrhythmic storm, or cardiac device dysfunction (e.g., pacemakers, defibrillators); cardiac surgery: patients experiencing cardiogenic or hypovolemic shock following valve replacements, aortic vascular surgeries, intracardiac and intravascular devices (e.g., intra-aortic balloon pump, assist devices), or those undergoing heart transplantation; sepsis: patients with septic shock due to bacteremia or autonomic dysregulation following conditions such as pancreatitis, hepatitis, pneumonitis, or pyelonephritis; other surgeries: patients with ventilatory needs, analytical complications, or secondary effects due to organ ischemia post-liver, lung, or hematopoietic transplantation, as well as those undergoing oncological surgeries of various organs; and miscellanea: patients not classifiable in the previous groups, including those with multi-organ failure and hemorrhagic shock post-surgery (e.g., gynecological, oncological, digestive), hydrostatic decompensation with elevated intra-abdominal pressure, acute respiratory distress syndrome, pharmacological intoxications, or polytrauma. 

Data were meticulously collected by nephrologist through a thorough review of electronic medical records, and a specific database was created for the study. The collected data included demographic information, patient functional and hemodynamic characteristics, blood analytical parameters, and hemodynamic status at CRRT initiation. Renal function evolution post-CRRT was documented at one, six, and twelve months after the end of CRRT. Patients were grouped according to their reason for ICU admission, with follow-up continued until twelve months post-CRRT or until the patient’s death.

The study was approved by the Ethics Committee (PI 151/24) of Hospital Universitario Puerta de Hierro Majadahonda, with a waiver of consent.

Continuous variables were presented as means (standard deviation, SD) or medians (interquartile range, IQR) and compared using the ANOVA or Kruskal–Wallis test depending on data distribution. Categorical variables were expressed as percentages and compared using the Chi-square test. A *p* value < 0.05 was considered statistically significant. Statistical analyses were performed using STATA 14.0 (Stata Statistical Software: Release 14. College Station, TX, USA: Stata Corp LP).

## 3. Results

### 3.1. Patient Description

**Figure 1:**
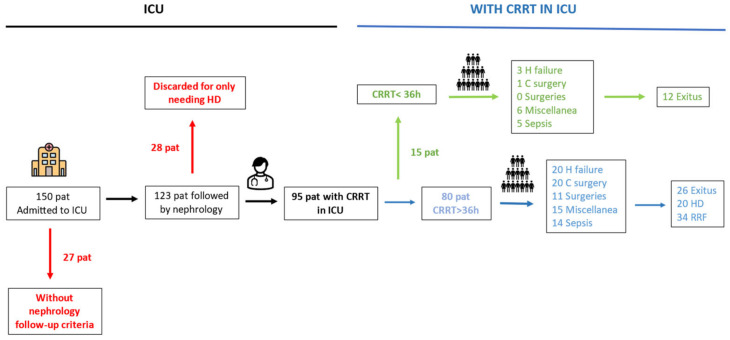
Flow chart of patients admitted to the ICU with AKI who required nephrology follow-up to assess the need for RRT. HD: conventional hemodialysis; CRRT: continuous renal replacement therapy; RRT: renal replacement therapy; H failure: heart failure; C surgery: cardiac surgery; RRF: residual renal function; Pat: patients; ICU: intensive care unit; and AKI: acute kidney injury.

During 2019, 123 patients admitted in the medical and surgical ICUs were evaluated by a nephrologist for CRRT. Of these, 95 patients met the inclusion criteria (Figure 1). Among them, 15 patients received CRRT for less than 36 h (CRRT < 36 h). All of these cases were initiated by on-call staff during the night or weekend. This group was predominantly male (10 men and 5 women), with an average age of 64.2 years (SD = 14.8) and a Charlson index of 6.5 (SD = 3.8). The primary reasons for ICU admission were sepsis (five cases), heart failure (three cases), and other causes in the remaining cases. CRRT was prescribed for AKI (nine cases of prerenal AKI, one case of ATN), sepsis (two cases), or refractory hemodynamic shock (three cases). Three patients had previous CKD. The majority of these patients (12 out of 15) died within the first few hours, and CRRT was withdrawn early in the other three patients after a structured multidisciplinary re-evaluation, with no further need for renal replacement therapy (RRT) (Figure 1). We did not identify a specific patient profile that distinguished this group from patients who required CRRT for more than 36 h (CRRT > 36 h).

The final data set for analysis comprised 80 patients who received CRRT for more than 36 h (CRRT > 36 h). This group was predominantly men (72.5%), with a mean age of 65.3 years (SD = 13.6) and a Charlson index of 6.4 (SD = 3.1). Previous CKD was present in 27.8% of the patients and was attributed to causes such as glomerulonephritis, diabetes mellitus, and other conditions (Table 1).

To better describe clinical phenotypes, patients were categorized into subgroups: heart failure (*n* = 20), cardiac surgery (*n* = 20), sepsis (*n* = 14), other surgeries (*n* = 11), and miscellanea (*n* = 15). Patients with heart failure admitted to the medical ICU were mainly males, were numerically the youngest, had the highest percentage of previous CKD (40%) and cardiac comorbidities, and were associated with the worst outcomes in the long-term follow-up (Table 1). 

The mean sCr for all groups at the start of CRRT was 2.3 mg/dL, mean serum urea (sUr) was 123.8 mg/dL, and urine output was less than 290 mL/d, despite an adequate use of diuretics and vasoactive agents at the initiation of CRRT. As a result, low urine output was a more significant factor than uremic status in the decision to start CRRT. The miscellanea subgroup had the worst laboratory values (sCr, sUr, lactate, and low bicarbonate), along with a lower urine output (Table 2).

Prerenal AKI was most common in heart failure (60%) and miscellanea groups (73.3%), while ATN was more prevalent in cardiac surgery (65%) and other surgeries (72.7%) groups. However, these differences were not statistically significant (*p* = 0.06). Hemodynamic conditions at the initiation of CRRT were worse in both surgical groups, with an increased need for vasoactive support (82.5%) and extracorporeal membrane oxygenation, although these differences were not statistically significant (Table 2). No dosage adjustments were recorded due to variability in medication based on the patient’s critical needs.

The duration of the first CRRT ranged from 2 to 32 days (median time = 7 days; IQR = [4–12]); patients in the surgeries group remained on CRRT for a median of 7 days, IQR = [5–19], while patients with sepsis had a median CRRT duration of 6 days, IQR = [4–6]. Continuous venovenous hemodiafiltration (CVVHDF) was the most commonly used CRRT modality across all subgroups and etiologies: 92% for prerenal AKI and 73% for hemodynamic control, with a higher percentage of slow continuous ultrafiltration (SCUF) used for hemodynamic control (17% vs. 8%). 

### 3.2. CRRT Transitions and Renal and Patient Outcomes

**Figure 2:**
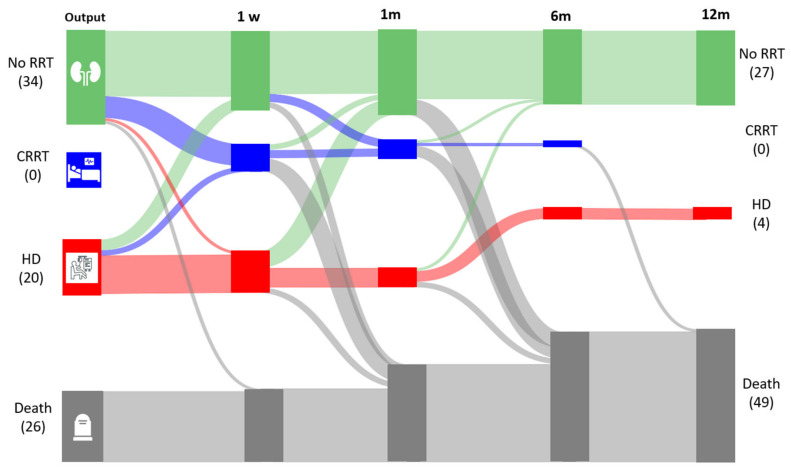
Sankey diagram including the transitions after the withdrawal of CRRT until the end of the follow-up. RRT: renal replacement therapy; HD: hemodialysis; CRRT: continuous renal replacement therapy; w: week; and m: month.

Upon the first discontinuation of CRRT, RRT therapy, renal function, and patient status were assessed at 7 days, 30 days, 6 months, and 12 months. This revealed significant variability in therapy transitions and outcomes, as summarized in Figure 2. These patterns included de-escalation from CRRT to conventional HD, new need for CRRT, requirement of chronic HD, renal recovery, or patient death.

Following the first CRRT, the renal function of 34 patients recovered, and 18 of these patients remained alive and free of dialysis at one year. An additional 9 patients required CRRT or HD at some point during the follow-up but were alive and no longer dependent on RRT at 6 and 12 months.

Twenty patients transitioned from CRRT to conventional HD. Of these, the residual renal function (RRF) of eight patients recovered within the first month. Seven patients remained on HD for one month and four patients for one year. Consequently, 13% of patients who survived the first year (4/31) remained on HD due to CKD. This group accounted for 9.5% of the incident patients that had undergone HD at our hospital [14].

Mortality was high, with 26 patients dying after CRRT and 49 within a year. Transitions between techniques and modalities were more frequent within the first week following CRRT withdrawal (Figure 2).

Recovery of RRF without the need for any RRT was similar in patients with prerenal AKI and hemodynamic control (39.5% vs. 41.9%). However, patients with prerenal AKI had a higher percentage of conventional chronic HD at discharge (31.6 vs. 19.4%) and slightly lower mortality (29% vs. 38.7%; *p* value = 0.9).

In the long-term follow-up, 35.5% of patients with refractory shock indication for CRRT recovered RRF, compared to 29% in those with prerenal AKI. Mortality increased from 1 to 6 months in all groups but remained stable from 6 to 12 months. 

**Figure 3:**
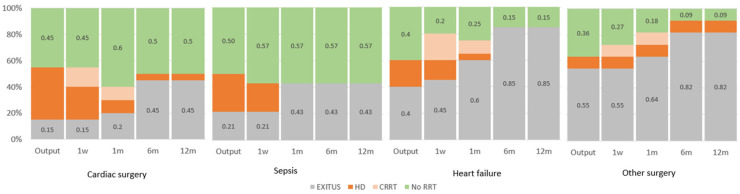
Mortality (%) and the evolution of the need for RRT (%) by patient subgroups in each time interval after EDT withdrawal in the ICU. CRRT: continuous renal replacement therapy; HD: hemodialysis; ICU: intensive care unit; and RRT: renal replacement therapy.

Patients with sepsis had the highest rate of RRF recovery without RRT (57% of all, and all of the alive patients) starting at one-month post-CRRT, followed by patients that had undergone cardiac surgery. The sepsis and cardiac surgery subgroups had the best survival rates across all follow-up stages, whereas the heart failure and miscellanea subgroups experienced increased mortality starting one-month post-CRRT, showing the highest mortality at one year (Figure 3).

The highest mortality at CRRT discontinuation occurs in the surgeries subgroup (54.5%), which also had the highest Charlson index (mean = 8.2; SD = 4.0). Conversely, the cardiac surgery subgroup had the lowest mortality and was the least comorbid. 

One-year post-CRRT, the heart failure and surgeries subgroups had the highest mortality and comorbidities, while sepsis and cardiac surgery subgroups had lower mortality and fewer comorbidities. 

## 4. Discussion

We analyzed the real-world experience of a nephrology consultant service with CRRT in CCU of a reference university hospital over one year. We categorized patients based on clinical profiles, etiologies, and CRRT indications. Our findings suggest that low urine output, refractory to conventional approach, was a more significant factor for CRRT initiation than uremic values. We observed substantial variability in clinical practices regarding transitions between different RRTs and noted poor short- and long-term prognosis for these patients. A notable subgroup of patients died within the first few hours of CRRT, likely representing a futile intervention in desperate situations. Additionally, we have obtained data on the long-term follow-up that highlights the impact of CRRT on the incidence to chronic HD programs, identifying areas for improvement in our local procedures and new research opportunities.

The study population closely mirrors the general ICU population with AKI requiring RRT, with a prevalence of 5–10% [15]. These patients are predominantly middle-aged men with diabetes, pre-existing CKD, and significant comorbidities. Both medical and surgical ICUs exhibited high in-hospital mortality (43.8% one-month post-CRRT), consistent with other studies reporting mortality rates of 30–70% [16,17]. 

Our study focused primarily on describing the reality of CRRT treatment in the ICU and its long-term outcomes. We found a high mortality rate of over 60% at 12 months and significant variability in transitions between techniques, differing from the trial protocols supporting clinical guidelines [11,12,13]. This variability in care practices and transitions should be individualized and considered in future study designs and analyses.

In our study, high mortality within six months and chronic HD dependence in survivors (13%) were observed, aligning with other studies showing over 50% short-medium-term mortality and 25% dialysis-free survival post-CRRT [13,14,15,16,17]. Patients whose renal function recovered without RRT showed better one-year survival rates compared to those dependent on HD, indicating that early mortality is more attributable to ICU admission reasons than the associated AKI.

A subgroup of patients received CRRT for just over 24 h, with more than 80% dying within those first hours. This urgent and hasty indication for CRRT, likely decided unilaterally by ICU on-call staff, deserves specific analysis in further studies and identifies an area for improvement. Early follow-up of patients at risk of AKI by the ICU specialist in charge of the patient and the dedicated consultant nephrologist during regular hours would be desirable. This approach would allow timely intervention, avoid futile prescriptions and, above all, outline an integrated plan adapted to the patient based on guidelines and criteria to de-escalate CRRT until potential recovery. The initiation RRT in ICU patients has been extensively studied and debated, particularly regarding its impact on patient outcomes. The IDEAL-ICU trial [18], a notable study, compared early versus delayed initiation of RRT in patients with septic shock and AKI. This trial was halted prematurely due to findings suggesting no significant difference in 90-day mortality between the two groups, indicating potential futility of early RRT initiation in such cases. Similarly, the AKIKI trial [19] investigated early versus delayed RRT initiation in patients with AKI. This study revealed that the delayed strategy reduced the number of patients requiring RRT without a notable increase in mortality rates. These outcomes underscore the importance of carefully evaluating the indication for RRT in critical care scenarios to avoid unnecessary interventions that may not improve patient survival.

AKI and volume overload are prevalent complications among critically ill ICU patients (with a prevalence of 5–10%) [20], leading to hemodynamic abnormalities, increased morbidity and mortality, adverse effects, and prolonged hospital stays. The decision to initiate CRRT early in patients with prerenal AKI stage 3 is preferred to prevent potential irreversible renal damage, as prerenal AKI often results from decreased renal blood flow due to factors such as dehydration, heart failure, or sepsis. CRRT allows for the controlled removal of waste products and excess fluids, is less aggressive than conventional hemodialysis, and also helps restore electrolyte balance and improve hemodynamics, thereby supporting subsequent renal recovery. This approach is supported by a meta-analysis which concluded that the early initiation of CRRT may be recommended regardless of renal function laboratory values in critically ill postoperative patients, as early administration of this therapy could reduce mortality rates and promote the recovery of baseline renal function in this patient population [21]. Similarly, a retrospective observational study [22] also found that delayed initiation of RRT might be associated with increased short-term mortality among critically ill ICU patients, recommending early initiation of CRRT in the presence of oligoanuria before the development of AKI 3.

CRRT is commonly used but has been shown to be an independent mortality factor in patients with AKI, even when admitted for other reasons. Despite advances in technology, mortality rates remain high [20,23,24].

Our findings suggest that morbidity could be associated with higher mortality immediately post-CRRT and at one year, without a clear relationship with age or the initial medical/surgical condition. This supports the observation of high short- to medium-term mortality rates, aligning with other studies that show only 21% survival at ten years [25,26]. 

We categorized AKI etiology and subdivided patients by ICU admission pathology, revealing a predominance of prerenal AKI. CVVHDF is the primary modality used in the ICU due to its slow, continuous nature, which prevents sudden changes in volume and electrolytes typical of HD. CVVHDF enhances gas exchange by reducing hydrostatic pressure and improving ventricular filling pressures, positively contributing to the overall management of critically ill patients beyond AKI [23,24,25,26]. However, a review by Valdenebro et al. [13] found no significant mortality, ICU/hospital stay, renal function recovery, or cardiovascular stability advantages of continuous techniques over intermittent techniques.

The heart failure subgroup did not have a higher percentage of previous heart failure, suggesting that these patients experienced their first episode of heart failure with secondary AKI during their ICU stay. This finding underscores the importance of careful volume management during early AKI to prevent aggravation of the condition. Open indications and transitions between different CRRT modalities offer significant advantages in terms of flexibility, treatment personalization, patient stability, and resource optimization. Transitioning from CVVHDF to CVVHD can be beneficial as the patient recovers adequate diuresis, and switching from CRRT to intermittent dialysis can be considered as renal function and laboratory results improve. Additionally, transitioning from CVVHD to SCUF may be necessary to manage fluid removal more effectively. These adaptations enhance clinical management and improve treatment outcomes. As nephrologists, we use SCUF electively in patients with volume overload (heart failure and cardiac surgery) to avoid renal function deterioration requiring immediate dialysis. Observational studies support SCUF’s effectiveness over intermittent HD in achieving negative fluid balance, making it essential for hemodynamic support in critically ill, volume-overloaded patients [20,23,24,25,26]. Early intervention could optimize the management of critically ill patients, proposing nephroprotection and AKI prevention strategies. Screening for and addressing factors like cardiopulmonary comorbidity, hemorrhage, hemolysis, toxin accumulation in sepsis, and nephrotoxic agent-induced oliguria is crucial. Primary prevention focuses on optimizing blood volume and hemodynamics to ensure renal perfusion, avoiding nephrotoxic drugs. Secondary prevention aims at early renal recovery through close monitoring of nephrotoxic complications, hyperglycemia, respiratory acidosis, and drug dosages [27].

Oligoanuria associated with prerenal AKI is mainly seen in the cardiac surgery subgroup. According to Mas-Font et al. [28], perioperative AKI oliguria results from water and salt retention in response to tissue damage, pain, and hypovolemia/hypotension, requiring controlled vasoactive support, intravenous fluids, transfusions, and analgesia. These parameters are critical “early action objectives” for preventing secondary kidney damage that worsens prognosis. 

We only recorded whether patients required vasoactive support, due to variability in medication dosing, which often changes almost hourly based on the patient’s critical needs. This highlights the characteristic instability of critically ill patients and justifies the use of continuous initiation therapies, which can then be transitioned to different modalities. A nephrologist supervises the prescribed CRRT regimen daily, assessing its necessity, maintenance, and renal function recovery post-withdrawal.

The miscellanea group presented the worst initial analytical data at CRRT onset, possibly due to its complexity, encompassing both medical and surgical pathologies. Delayed nephrology consultation might worsen clinical and analytical status, but post-CRRT, these patients did not have the highest mortality. This aligns with Chou et al. [29], who through propensity score analysis, determined that the RIFLE criteria poorly predict the benefits of early or late initiation of RRT in critically ill patients.

Risk factors for non-recovery of renal function post-AKI include advanced age, previous CKD, and comorbidities [30,31]. This was confirmed in our study, where patients with CKD were more likely to remain on HD. Evaluating discharge time from CRRT was proposed, even though ICU discharge timing might have been more relevant. This study focuses on identifying parameters upon ICU admission that condition CRRT need, determine CRRT termination, or influence post-AKI renal recovery. CKD establishment with or without RRT is analyzed to develop nephroprotection strategies involving nephrologists in the multidisciplinary critical care team [32].

External validity limitations include differences in hospital service portfolios, pathologies, and available techniques. Our results from a hospital with complex surgical and transplant programs may not represent other ICUs at different care levels.

## 5. Conclusions

Management of ICU patients with AKI requiring CRRT is complex due to the variability in indications, comorbidities, and transitions between modalities. All of these factors make it challenging to analyze own results and compare them with clinical trials. High in-hospital mortality and persistent dialysis needs are common among those who survive and get discharged from the ICU. In fact, it constitutes its own category as a cause of chronic RRT, which is often forgotten. 

The reasons for ICU admission play a crucial role in the evolution of AKI, with the patients in the sepsis and cardiac surgery groups demonstrating better survival outcomes. Given this complexity, a multidisciplinary approach involving ICU and nephrology teams is essential, with continuous, around-the-clock monitoring and collaboration to ensure effective care planning and improve patient outcomes. 

## Figures and Tables

**Table 1:** Baseline demographic data according to the cause of admission to the ICU.

	Heart Failure	Cardiac Surgery	Other Surgeries	Sepsis	Miscellanea	Total	*p* Value
N	20	20	11	14	15	80	
Men (%)	95.0	55.0	63.3	64.3	80.0	72.5	0.05
Age (year)	62.7(15.5)	69.1 (9.8)	65.3 (12.1)	66.0 (13.7)	62.9 (16.7)	65.3 (13.6)	0.6
Charlson index	7 (3.4)	5.5 (1.9)	8.2 (4.0)	5.4 (2.5)	6.7 (3.4)	6.4 (3.1)	0.09
Previous CKD (%)	40.0	20.0	27.3	23.1	26.7	27.8	0.7
DM (%)	55.6	11.1	36.4	28.6	28.6	32.0	0.08
Ischemic heart disease (%)	40.0	31.6	27.3	14.4	13.3	26.6	0.3
Reduced LVEF (<50%) (%)	42.1	17.6	30.0	12.5	16.7	25.8	0.4
Diastolic dysfunction (%)	16.7	11.8	0.0	0.0	8.3	9.5	0.8
Previous H failure (%)	65.0	85.0	45.5	78.6	53.3	67.5	0.1
Stroke or TIA (%)	10.0	20.0	18.2	0.0	13.3	12.5	0.5
Liver disease (%)	15.0	10.0	54.5	28.6	40.0	26.2	0.04
Previous neoplasia (%)	25.0	15.0	45.5	28.6	33.3	27.5	0.5
COPD (%)	40.0	20.0	36.4	14.3	26.7	27.5	0.4
Exitus/CRRT output (%)	40.0	15.0	54.2	21.4	40.0	32.5	0.4
Exitus by 1 year (%)	85.0	45.0	81.8	42.9	53.3	61.3	0.4

Data are expressed as percentages, means, and standard deviation according to the analyzed variables. CKD: chronic kidney disease; DM: mellitus diabetes; LVEF: left ventricular ejection function; COPD: chronic obstructive pulmonary disease; TIA: transient ischemic attack; CRRT: continuous renal replacement therapy; and ICU: intensive care unit.

**Table 2:** Etiology, hemodynamic situation, and modality at onset of CRRT according to cause of admission.

	Heart Failure	Cardiac Surgery	Other Surgeries	Sepsis	Miscellanea	Total	*p* Value
Etiology							0.06
Prerenal AKI (%)	60.0	35.0	27.3	35.7	73.3	47.5	
ATN (%)	40.0	65.0	72.7	64.3	26.7	52.5	
Laboratory data							
sCr (mg/dL)	2.5 (1.6)	2.0 (1.1)	2.1 (0.9)	2.1 (1.4)	3.1 (1.4)	2.3 (1.3)	0.1
sUr (mg/dL)	137.3 (66.8)	113.2 (67.3)	104.8 (60.7)	115.5 (46.3)	143.2 (77.4)	123.8 (65.6)	0.45
Bicarbonate (mmol/L)	21.6 (4.3)	25.7 (4.2)	22.5 (2.6)	24.3 (5.8)	19.7 (6.5)	22.9 (5.2)	0.006
Lactate (mmol/L)	1.4 [1–4]	1.1 [0.8–1.6]	1.6 [1.3–1.9]	1.4 [1.1–2.4]	2.2 [1.3–6.8]	1.4 [1–3]	0.8
Diuresis (ml/h)	4.6 (10.0)	7.2 (17.1)	13.8 (34.1)	38.8 (73.4)	0.9 (2.6)	11.8 (36.0)	0.002
ECMO (%)	20.0	15.6	18.1	0.0	1.0	12.7	0.4
VAS (%)	85.0	90.0	81.8	78.6	73.3	82.5	0.8
CRRT modality							0.3
CVVHDF (%)	85.0	75.0	81.4	85.7	100.0	85.0	
CVVHF (%)	0.0	10.0	0.0	14.3	0.0	5.1	
SCUF (%)	15.0	15.0	18.2	0.0	0.0	10.0	

Data are expressed as percentages, means, and standard deviation or median [IQR] depending on the analyzed variables. AKI: acute renal failure; ATN: acute tubular necrosis; VAS: vasoactive support; CVVHDF: continuous venovenous hemodiafiltration; CVVHF: continuous venovenous hemofiltration; SCUF: slow continuous ultrafiltration; CRRT: continuous renal replacement therapy; ECMO: extracorporeal membrane oxygenation; sCr: serum creatinine; and sUr: urea.

## Data Availability

The basic data set is provided, and the full data set can be provided for further analysis after a formal request is made to the corresponding author.
